# Olympic Ice Sports: A Narrative Review and Perspectives Toward Milano‐Cortina 2026

**DOI:** 10.1111/sms.70213

**Published:** 2026-02-18

**Authors:** Billy Sperlich, Chiara Zoppirolli, Florentina Hettinga, Lindsay Slater Hannigan, Steffi Colyer, Jeppe F. Vigh‐Larsen, Alessandro Fornasiero, Hans‐Christer Holmberg

**Affiliations:** ^1^ Integrative and Experimental Exercise Science and Training University of Würzburg Würzburg Germany; ^2^ CeRiSM (Research Centre of Mountain Sport and Health) University of Verona Rovereto Italy; ^3^ Department of Neuroscience, Biomedicine and Movement University of Verona Verona Italy; ^4^ Department of Human Movement Sciences Faculty of Behavioural and Human Movement Sciences, Vrije Universiteit Amsterdam Amsterdam the Netherlands; ^5^ Department of Physical Therapy University of Illinois at Chicago Chicago Illinois USA; ^6^ Department for Health University of Bath Bath UK; ^7^ Department of Movement and Sports Sciences, Faculty of Medicine and Health Sciences Ghent University Ghent Belgium; ^8^ School of Kinesiology University of British Columbia Vancouver British Columbia Canada; ^9^ Division of Machine Elements Luleå University of Technology Luleå Sweden; ^10^ Department of Physiology and Pharmacology, Biomedicum C5 Karolinska Institutet Stockholm Sweden

**Keywords:** gliding sport, ice sport, Olympic sport, winter sport

## Abstract

As the Milano‐Cortina 2026 Winter Olympics approach, a consolidated understanding of performance determinants across the diverse spectrum of ice sports is crucial, yet the scientific literature remains unevenly distributed. This structured narrative review synthesizes available evidence on key performance‐determining factors and contemporary training characteristics for Olympic ice sports, based on topic‐driven literature searches and qualitative synthesis. Disciplines are grouped according to their primary performance demands. (1) High‐volume gliding sports (long‐ and short‐track speed skating): Performance balances biomechanical efficiency (e.g., aerodynamic posture) against physiological constraints. This necessitates high annual training volumes (900–1100 h·year^−1^), polarized, mixed‐modal training, with short‐track adding critical tactical and pack‐dynamic elements. (2) Exposure‐driven gravity sports (bobsleigh, skeleton, luge): Performance is overwhelmingly determined by start velocity, with the initial 15–65 m contributing disproportionately to overall race outcome. Bobsleigh and skeleton training mirrors sprint athletes, prioritizing lower‐body power, while luge demands explosive upper‐body strength. (3) Arena‐based sports (ice hockey, figure skating, curling): These sports show varied demands. Ice hockey requires managing high‐intensity intermittent efforts, with 40%–50% of on‐ice distance performed at high skating speeds; figure skating hinges on the power and precision of high‐value jumps (e.g., triple and quadruple rotations); and curling relies on delivery accuracy and sweeping strength‐endurance. Sex‐specific differences, often related to absolute power output (skating, sliding) and biomechanics, are evident, although evidence remains limited or uneven across several disciplines. Rather than providing prescriptive training models, this review identifies discipline‐specific training priorities and key gaps in the current evidence base relevant to athlete preparation for Milano‐Cortina 2026.

## 1 Introduction

Since the first Winter Olympic Games in 1924, ice‐based sports have evolved from natural‐ice competitions into highly specialized disciplines shaped by advances in equipment, ice preparation, and training science, providing a foundation for contemporary performance analysis. Crucially, success in all disciplines is predicated on the fundamental mechanics of ice locomotion, particularly propulsion and gliding on a low‐friction interface.

### 1.1 Historical Development of Olympic Gliding Disciplines

Long‐track speed skating and bobsleigh have featured since the first Winter Olympics in 1924, with women's events added progressively from the mid‐20th century onward. Short‐track speed skating entered the Olympic program in 1992, while luge and skeleton were included later, with skeleton reintroduced in 2002. Arena‐based sports followed a different trajectory: figure skating moved from the Summer to the inaugural Winter Games in 1924, ice hockey has been included since 1924 (women's events since 1998), and curling became a regular medal sport in 1998.

The expansion of disciplines and women's events reflects both technical innovation and social progress, with women's medal events increasing from < 20% in 1960 to nearly 50% by 2022. Despite this growth, research remains unevenly distributed, with most evidence concentrated in endurance‐oriented ice sports and limited data available for sliding and arena‐based disciplines.

### 1.2 Shared Mechanics and Distinct Performance Demands

All Olympic ice sports share the fundamental challenge of generating and maintaining propulsion on a low‐friction surface while minimizing aerodynamic and mechanical losses. Success depends on the effective integration of muscular force, technical precision, and control of athlete–equipment dynamics. However, the contribution of metabolic, mechanical, and neuromuscular systems differs between disciplines (Table 1). Importantly, this classification reflects dominant performance constraints and is not intended to imply uniformity within each category; substantial within‐group variability exists, particularly between long‐ and short‐track speed skating, and is explicitly addressed in the sport‐specific sections.

**TABLE 1:** Overview of competition formats, venues, sex categories, and key performance metrics across Olympic on‐ice disciplines.

Sport event	Format	Venue	Sex	Performance metric	Men	Women
Long‐track speed skating 500 m	TT	IO‐S	♂, ♀	World Record (s)	33.61	36.36
Long‐track speed skating 1000 m	TT	IO‐S	♂, ♀	World Record (min:s)	1:05.37	1:11.61
Long‐track speed skating 1500 m	TT	IO‐S	♂, ♀	World Record (min:s)	1:40.17	1:49.83
Long‐track speed skating 3000 m	TT	IO‐S	♀ only	World Record (min:s)	—	3:52.02
Long‐track speed skating 5000 m	TT	IO‐S	♂, ♀	World Record (min:s)	6:01.56	6:39.02
Long‐track speed skating 10 000 m	TT	IO‐S	♂ only	World Record (min:s)	12:25.69	—
Long‐track speed skating mass start	TT	IO‐S	♂, ♀	Race time (min:s)	≈7:45–8:15	≈8:15–9:00
Long‐track speed skating team pursuit	TT	IO‐S	♂, ♀	World Record (min:s)	8 laps (3200 m) 3:33.66	6 laps (2400 m) 2:50.76
Short‐track speed skating 500 m	HE	IR‐S	♂, ♀	World Record (s)	39.505	41.416
Short‐track speed skating 1000 m	HE	IR‐S	♂, ♀	World Record (min:s)	1:20.875	1:25.958
Short‐track speed skating 1500 m	HE	IR‐S	♂, ♀	World Record (min:s)	2:07.943	2:14.354
Short‐track speed skating 3000 m relay	HE	IR‐S	♀ only	World Record (min:s)	—	4:02.809
Short‐track speed skating 5000 m relay	HE	IR‐S	♂ only	World Record (min:s)	6:28.625	—
Short‐track speed skating mixed team relay	HE	IR‐S	♂ + ♀	World Record (min:s)	2:35.339	
Figure skating singles	PR	IR‐S	♂, ♀	Max rotations (Total Score WR)	4.5 (335.30)	4.0 (272.71)
Figure skating pairs	PR	IR‐S	♂ + ♀	Max throw jump rotations	3.5–4	3–4
Figure skating ice dance	PR	IR‐S	♂ + ♀	Element levels/GOE	—	—
Figure skating team event	TA	IR‐S	♂ + ♀	Aggregate points	—	—
Luge singles	MR	ST‐S/M	♂ + ♀	Top speed km·h^−1^	130–145	125–140
Luge doubles	MR	ST‐S/M	♂, ♀	Top speed km·h^−1^	135–150	130–145
Luge Team Relay	MX	ST‐S/M	♂ + ♀	Top speed km·h^−1^	130–145	125–140
Bobsleigh 2‐man/woman	MR	ST‐S/M	♂ + ♀	Top speed km·h^−1^	135–150	—
Bobsleigh 4‐man	MR	ST‐S/M	♂ only	Top speed km·h^−1^	135‐150	
Bobsleigh monobob	MR	ST‐S/M	♀ only	Top speed km·h^−1^	120‐135	125–140
Skeleton singles	MR	ST‐S/M	♂, ♀	Top speed km·h^−1^	135–150	125–140
Skeleton mixed team	MX	ST‐S/M	♂ + ♀		130‐150	
Curling team	TA	IR‐S	♂, ♀	Shot success %	80%–85%	75%–82%
Curling mixed doubles	TA	IR‐S	♂ + ♀		80%–85%	
Ice hockey tournament	HE	IR‐S	♂ + ♀	Max skating/shot speed	37 km·h^−1^; 165 km·h^−1^	33 km·h^−1^; 120 km·h^−1^

Abbreviations: ♀, female; ♂, male; ♂ + ♀, mixed‐sex event; GOE, grade of execution; HE, head‐to‐head; IO‐S, indoor oval skating rink; IR‐S, indoor ice rink; MR, multiple runs; MX, mixed format; PR, program‐based (judged routine); ST‐S/M, sliding track (synthetic or mixed natural ice); TA, team aggregate; TT, time trial.

To describe these differences, this review applies three classifications based on the dominant performance demand. This framework groups disciplines based on shared physiological and mechanical limiting factors, enabling more meaningful cross‐sport comparisons.

1. High‐volume gliding sports (long‐ and short‐track speed skating) depend primarily on aerobic and anaerobic energy turnover, biomechanical efficiency, and pacing regulation. Performance requires high training volume, refined movement economy, and the ability to sustain aerodynamic posture under fatigue.
2. Exposure‐driven gravity sports (bobsleigh, skeleton, luge) rely on rapid acceleration and precise aerodynamic control during short, high‐speed descents. Performance depends on start effectiveness, neuromuscular power, and the coordination of steering or gliding technique.
3. Arena‐based ice sports (ice hockey, figure skating, curling) combine intermittent or fine‐motor demands with complex cognitive, technical, and tactical components. These disciplines integrate power, skill execution, perceptual–motor control, and strategic decision‐making.

The classification of Olympic ice sports into high‐volume gliding, exposure‐driven gravity, and arena‐based disciplines was not derived from a formal systematic algorithm, but emerged iteratively as a conceptual framework during evidence synthesis. This framework is based on the dominant physiological, biomechanical, and technical constraints identified across disciplines and is intended to support cross‐sport comparison rather than imply homogeneity within categories. The upcoming Milano–Cortina 2026 Winter Olympic Games highlight the need to consolidate the scientific evidence on performance determinants in Olympic ice sports. Although individual studies describe aspects of physiology, biomechanics, and training, the overall knowledge base remains fragmented across disciplines and uneven with respect to sex‐specific data. This narrative review aims to:

(i) integrate current findings on the physiological, biomechanical, and technical factors that determine performance across all Olympic ice sports; (ii) describe contemporary training structures within each category of performance demand; and (iii) identify key research gaps relevant to optimizing athlete preparation for 2026.

By comparing disciplines within a unified performance framework, the review links mechanistic understanding with applied relevance for sport scientists, coaches, and practitioners involved in high‐performance ice sport preparation.

## 2 Method

This narrative review was prepared as part of a multi‐paper special issue addressing scientific and applied topics relevant to preparation for the Olympic Winter Games Milano–Cortina 2026. It was designed as an integrative, concept‐driven narrative synthesis to complement discipline‐specific contributions, rather than as a systematic or reproducible evidence‐mapping exercise.

### 2.1 Literature Search Strategy

Structured, topic‐driven literature searches were conducted in PubMed/MEDLINE, Web of Science, and Google Scholar between April and October 2025. Searches combined keywords related to Olympic ice sports and elite performance (e.g., endurance training, training load, strength and power, recovery, technology, athlete health), alongside sport‐specific terms (e.g., speed skating, ice hockey, bobsleigh, figure skating, curling). Search strings were adapted to each database and refined iteratively to balance sensitivity and relevance.

The search process was iterative and adaptive, with thematic emphases refined as the conceptual framework of dominant performance demands evolved. Reference lists of key reviews, consensus statements, and position papers were screened using backward and forward citation chaining to identify additional relevant literature.

### 2.2 Study Selection and Eligibility Criteria

We prioritized peer‐reviewed English‐language studies published between 2005 and 2025, while including seminal earlier work where conceptually foundational. Eligible studies primarily involved athletes classified as “highly trained/national level” to “World Class.” Studies involving untrained, pediatric, or clinical populations without direct relevance to elite performance were excluded. Consistent with the narrative intent of the review, no formal study‐count thresholds, screening flow diagram, or reproducibility metrics were applied.

Given the uneven distribution of evidence across ice sports, a broad range of study designs (experimental, observational, modeling, and applied case studies) was included. Where discipline‐specific data were sparse, applied reports and mechanistic evidence from related sports were selectively integrated, with such extrapolations explicitly acknowledged. Study selection was guided by relevance to performance determinants, training characteristics, and applied implications.

### 2.3 Evidence Synthesis

Literature was organized into three domains: (1) performance determinants and training methodologies; (2) recovery, health, and fatigue‐related factors; and (3) technology and applied innovations. Findings were synthesized narratively to identify shared and divergent physiological, biomechanical, and technical constraints across ice sports, with disciplines grouped by dominant performance demands to support cross‐sport comparison. Narrative synthesis was selected due to substantial heterogeneity in performance metrics, study designs, and outcome variables, and the strength and limitations of the evidence were considered where relevant.

### 2.4 Methodological Considerations and Limitations

Consistent with its narrative nature, no systematic search protocol, risk‐of‐bias assessment, or meta‐analysis was performed, and the review is not intended to be exhaustive. Potential limitations include language bias, selective inclusion of applied evidence, and reliance on extrapolation in disciplines with limited ice sport–specific data.

The evidence base is substantially stronger for high‐volume, endurance‐limited ice sports than for exposure‐driven or arena‐based disciplines, where endurance primarily serves a supportive or feeder function. Accordingly, the depth of analysis across sports is uneven, reflecting current research availability rather than differential importance. These gaps are explicitly highlighted and framed as priorities for future research.

## 3 High‐Volume Gliding Sports (Long‐ and Short‐Track Speed Skating)

Although long‐ and short‐track speed skating are grouped as high‐volume gliding sports based on shared constraints related to posture, drag, and sustained force production, they differ markedly in athlete phenotype and dominant performance determinants [1]. Long‐track speed skating, particularly at middle and long distances, places a greater emphasis on aerobic energy turnover [2], pacing skills that develop throughout adolescence [3], and the ability to sustain an efficient crouched posture under prolonged fatigue while maintaining technical ability [4, 5]. In contrast, short‐track speed skating imposes higher relative demands on anaerobic power, rapid accelerations, tactical decision‐making, and pack dynamics, with performance outcomes strongly influenced by positioning and situational context [6]. Recognizing these within‐group distinctions is essential for translating the classification into discipline‐specific training and coaching practice.

### 3.1 Long‐Track Speed Skating a factor distinguishing faster from slower athletes

In long‐track speed skating, a large part of the skater's mechanical power output is used to overcome energy losses to air and ice friction, with aerodynamic drag being the dominant resistive force at racing speeds (> 45 km·h^−1^) [7, 8]. Reducing aerodynamic drag is therefore critical and is achieved by reducing frontal area through the characteristic crouched position. This posture yields a smaller pre‐extension knee angle, a more horizontal trunk, and an associated smaller push‐off angle, reflecting a more effective push‐off [7, 9, 10]. The ability to maintain this characteristic crouched posture throughout the race thus seems a key performance differentiator, as was also suggested in junior elite athletes [5]. With fatigue, posture typically deteriorates, where the pre‐extension knee and push‐off angles increase, impairing both aerodynamics and push‐off effectiveness [8, 9]. This degradation is closely associated with velocity declines, particularly via the increase in push‐off angle during longer events [9]. In junior elite athletes, development over competitive seasons showed a shift toward smaller push‐off angles and a better ability to prevent a decay in posture throughout the race [5], a factor distinguishing faster from slower athletes.

Physiologically, the benefits associated with this biomechanically favorable crouched position also create a challenge: the posture substantially lowers frontal area and drag, but also reduces muscle blood flow to the thigh [11], constraining oxygen delivery and reinforcing the challenge of preserving effective technique under fatigue [10, 12]. A rapid desaturation occurs directly after the start of a race [13], with an asymmetry in muscle oxygenation between both legs associated with skating through the curves [11]. Additionally, evidence indicates that speed skating technique affects oxygenation, with consequences for fatigue and recovery kinetics [14]. Managing this interplay between biomechanical and physiological factors, and finding the optimal trade‐off, appears to be an important determinant of speed skating performance.

Another performance‐determining skill specific to speed skating, driven by the high aerodynamic drag forces, emerges in the team events, where drafting is critical. Synchronization can reduce aerodynamic drag by up to ~25% in wind‐tunnel tests, translating into ~3%–5% lower physiological intensity (heart rate)—underscoring the value of coordinated pacing strategies [15, 16]. Skaters in subsequent drafting positions also show benefits in both heart rate and ratings of perceived exertion [16]. While coaches and athletes recognize these benefits, they acknowledge limited knowledge or experience regarding how to optimize drafting skills through training [17]. Since in speed skating, team disciplines are relatively new to the Olympic agenda, this will be an interesting area for further research.

Elite long‐track speed skaters typically perform ~800–900+ hours annually, following a polarized, mixed‐modal approach with ~75%–80% of endurance training at low intensity. This structure reflects the sport's unique physical demands: the deep, muscularly demanding skating posture and high mechanical load make long sessions challenging and limit specific on‐ice training to ~150–200 h per year. Therefore, the vast majority of low‐ and moderate‐intensity sessions are performed as cross‐training (especially cycling), while the limited on‐ice time is prioritized for higher, competition‐specific intensities and racing [18]. Endurance work is supplemented by strength and power training (lower body/core) and dryland skating simulations [19, 20].

### 3.2 Short‐Track Speed Skating

Unlike long‐track's time‐trial format, short track is a rank‐based, head‐to‐head sport in which outcomes are shaped by starting position, pack dynamics, and round‐by‐round advancement (International Skating Union (ISU) 2018/2021 [21]. Performance rewards positioning: inside‐lane starts raise win probability (~28% in 500/1000 m; 22% in 1500 m), and pacing variability increases with distance, implicating energy conservation and flexible tactics [21]. Recent data confirm that elite skaters dynamically adjust their pacing in response to situational factors such as heat stage, number of opponents, sequential effects, and qualification opportunities—especially during the opening laps—highlighting the interaction between competitive context and energy distribution [22, 23, 24]; see also “Mind‐Muscle‐Environment Interactions: Psycho‐physiological Determinants of Optimal Pacing in Olympic Winter Endurance Sports” in this topical issue).

Curves impose substantial inward‐force demands and require crucial curve‐specific technique [25, 26]. Technically, faster laps are associated with a deep, stable posture (e.g., greater knee/hip flexion, lower pelvis) that enables efficient force application in the curves, characterized by a rearward center of pressure and lower lateral peak forces [25, 26]. This posture is associated with restricted quadriceps blood flow and rapid oxygen desaturation during short‐track races [27]. While men generally record faster laps, consistent with larger body size and strength, the primary technical determinants are shared across sexes; women tend to benefit more from deeper knee flexion and a lower turn posture, whereas men's marginal gains are more linked to optimizing ankle control [26].

While short‐track skaters also develop a robust endurance base, often through high‐volume, mixed‐modal training, this similarity to long‐track speed skating is largely confined to foundational aerobic preparation [19]. Consequently, descriptions of short‐track training are often inferred from related endurance disciplines and long‐track speed skating, particularly with respect to the development of a general aerobic base. Importantly, this inference should not be interpreted as equivalence: short‐track speed skating places substantially greater emphasis on repeated accelerations, anaerobic power, tactical decision‐making, and pack dynamics than long‐distance long‐track events [13, 28]. Also, the stronger asymmetric demands in short‐track events that affect blood flow impact on oxygenation, perceived fatigue and recovery [13].

Overall conditioning often involves substantial off‐ice cross‐training (e.g., cycling), a strategy employed because the crouched, locally demanding posture—which can reduce muscle blood flow—limits the tolerable volume of specific on‐ice sessions [10, 28]. This endurance foundation is supplemented by strength and power training to develop essential strength qualities and dryland skating simulations [10, 28, 29].

The primary divergence from long‐track is on‐ice, where a large portion of time is spent mastering pack dynamics (e.g., drafting, overtakes) [23]. Furthermore, informed by race analyses showing strong effects of starting position and greater pacing variability at longer distances, short‐track interval training commonly emphasizes race‐simulation, rehearsing scenarios like explosive starts for lane positions and accelerations to break from the pack [21]. This continual focus on reactivity, decision‐making under pressure, and energy conservation underpins the tactical layer that distinguishes short‐track demands [22].

Compared with long‐track speed skating, particularly at middle and long distances, short‐track speed skating places a greater relative emphasis on neuromuscular power, rapid force development, and strength‐related qualities. These demands arise from frequent accelerations, explosive starts, and repeated tactical repositioning within pack‐based racing, which increase the importance of lower‐body strength and power training alongside endurance development [13, 30, 31]. While the literature has not established major sex‐specific differences in overall training of elite short‐track skaters, modeling studies in elite short‐track skaters suggest that women may recover from and adapt to given training loads more rapidly than men, although this evidence remains indirect and model‐based [29, 32].

## 4 Exposure‐Driven Gravity and Technical Sports

### 4.1 Sliding Sports (Luge, Bobsleigh, and Skeleton)

Sliding sports involve athletes descending a steep, twisting ice track on a sled at high speed. While the glide phase (where athletes aim to minimize drag and find the optimal line) is crucial, a fast initial start (15–65 m from the starting block) is generally required to be successful in these sports. The velocity achieved in the first few seconds is a major contributor to the final race time, as there are limited or no opportunities for further propulsion after loading the sled (bobsleigh, skeleton) or ceasing the “paddling” phase. However, the start–finish time relationship may vary with track characteristics and driving demands [33], so start quality should be interpreted in that context. Nonetheless, a fast start is widely considered to be a prerequisite for success in sliding sports, and consequently, the biomechanics of the start and the physical preparation that underpins it are the primary focus of research and training.

In bobsleigh and skeleton, start performance is predominantly determined by an athlete's ability to sprint horizontally while overcoming the inertia of the sled. Key performance indicators are therefore closely related to those of elite sprinters, including high lower‐limb power and the effective application of horizontal force against the ground [34, 35]. During the bobsleigh push, propulsion is hip‐extensor dominant during early acceleration, with the ankle contribution increasing as velocity rises; this phase‐dependent pattern directly informs strength–power emphasis [35]. Notably, bobsleigh athletes tend to be taller and heavier [31] than both skeleton [30, 35] and luge [33] athletes, reflecting the advantage of greater total system mass for accelerating the heavier sled. Skeleton athletes, while lighter than bobsleigh athletes, also display a predominantly mesomorphic physique [36]. Off‐ice physical tests are highly predictive of skeleton start ability: a combination of 15‐m sprint time, countermovement jump height, and leg‐press power at high velocities explains ≈86% of the variance in sled velocity [37]. Skeleton start‐velocity profiling also shows that both pre‐load acceleration (loading the sled earlier with higher velocity) and loading phase quality (maximizing the velocity increase and minimizing the velocity drop during the loading phase) independently shape start performance, reinforcing a dual focus on, and interplay between, accelerative ability and load technique.

Unlike the running starts of bobsleigh and skeleton, the luge start is a seated, explosive upper‐body movement. The athlete initiates acceleration by pulling against two fixed handles and then paddling rapidly against the ice with spiked gloves [38, 39]. Consequently, start performance is strongly correlated with upper‐body strength and power. Specifically, one‐repetition maximum (1RM) values in the bench press and prone row, along with acromion‐olecranon length, strongly correlate with start times in elite luge athletes [38]. Furthermore, force‐velocity profiling indicates that an athlete's theoretical maximal velocity is a strong predictor of faster on‐ice start performance [39]. Taken together, these data demonstrate distinct phenotypic profiles across the sliding sports: bobsleigh athletes are generally the tallest and heaviest, skeleton athletes exhibit a sprinter‐like lower‐body power profile, and luge athletes display high upper‐body strength demands.

Given the critical importance of the start, training for sliding sports is overwhelmingly focused on developing explosive strength and power, with a clear distinction between the disciplines. For bobsleigh and skeleton, off‐ice conditioning mirrors that of a track and field sprinter. Training programs are built around heavy (high‐load) resistance strength training (e.g., squats), plyometrics to enhance explosive power, and high‐velocity sprint training [37, 40]. On‐ice and dry‐land push tracks are used to transfer these physical qualities to the specific skill of pushing the sled from a crouched position, using start‐velocity profiling to monitor progress and refine technique [34]. For luge, training prioritizes the development of the upper‐body musculature used in the pulling and paddling motion. The regimen is centered on heavy resistance exercises for the upper back, chest, and shoulders, such as prone rows, bench presses, and weighted pull‐ups [38], supplemented by simulators and refrigerated ramps to perfect the highly technical start sequence [39].

The greater absolute upper‐ and lower‐body strength and power of men, alongside their greater potential [41] typically results in faster start times and higher initial sled velocities across all three sliding sports [34, 40, 42]. In skeleton, however, the sleds of male athletes tend to be heavier than those of females and thus greater inertia must be overcome. Despite this, men's start times do tend to be faster than those of their female counterparts, reflective of the greater strength and power of the male athletes relative to the mechanical demands of the sport. Indeed, male athletes exhibit significantly faster resisted and unresisted sprint times and higher jump heights than female athletes, which are strongly correlated with their superior start performance [37, 40].

## 5 Arena‐Based Ice Sports

### 5.1 Ice Hockey

Ice hockey's high‐intensity intermittent nature, characterized by brief, repeated on‐ice shifts of ~30–80 s interspersed with 2–5 min rest, imposes substantial demands on both aerobic and anaerobic energy systems (~15–25 min total effective time on ice per game) [43]. Elite male players typically cover 4–6 km in total, depending on playing position and time on ice, with up to 50% of total distance performed at high skating speeds (> 17 km·h^−1^), alongside numerous intense, brief acceleration/deceleration movements [43]. Total distance covered (~4.5 km) and the proportion of distance skated at high speeds (~50%) are slightly lower in females, consistent with sex‐based differences in peak skating speeds, which reach ~29 km·h^−1^ and ~32 km·h^−1^ in females and males, respectively [44]. Forwards accumulate more high‐intensity skating and accelerations than defenders, attained in shorter on‐ice shifts, whereas the latter sustain greater total ice time and low‐to‐moderate workloads (total skating distance) [44, 45]. Success in matches, especially for forwards, is correlated with the ability to maintain high skating intensity (e.g., higher explosive ratio, % high‐force strides), with these intensity metrics tending to decline across periods [46]. Accordingly, ice hockey performance is characterized by anaerobic dominance during brief intense efforts, with aerobic metabolism serving a critical permissive role in sustaining repeated high‐intensity output across a full shift and across periods.

The intense on‐ice activities elicit marked metabolic disruptions, as evidenced by elevated muscle lactate concentrations and phosphocreatine depletion after shifts, and by ~60%–70% of both fast‐ and slow‐twitch fibers being classified as glycogen‐depleted (< 25% glycogen staining intensity compared to mean levels) following games, alongside impaired repeated‐sprint ability [47, 48] and increased ratings of perceived exertion. The ability to resist fatigue during severe metabolic and ionic perturbations is therefore critical, necessitating training simulating repeated high‐intensity sprint skating sequences (i.e., speed endurance training or repeated sprint training).

The aerobic system plays a key role in supporting anaerobic metabolism during supramaximal efforts and for/in recovery between repeated sprints [49]. This is reflected in relatively high maximal oxygen uptake values in ice hockey players (~55–60 mL·kg^−1^·min^−1^), which should not be interpreted as evidence of aerobic dominance during individual shifts, but rather as an indicator of the aerobic system's capacity to support repeated high‐intensity efforts and accelerate recovery between shifts. Moreover, intermittent aerobic exercise capacity distinguishes levels of play across national standards [50] and differentiates top‐ and bottom‐tier teams [51]. A mixed approach, emphasizing both aerobic and anaerobic conditioning, typically in different intermittent game‐based scenarios, is therefore important to stimulate beneficial physiological adaptations across metabolic systems.

For women, off‐ice physiological qualities transfer to their skating performance on‐ice, linking lower‐body power, aerobic capacity, and body composition (higher lean mass and lower fat mass) to both acceleration and speed [52, 53, 54]. Given the similarity between the acceleration phase of skate sprinting and sprint running, off‐ice speed work transfers most effectively to the initial acceleration phase, whereas maximal skating speed improvements require more specific on‐ice work [55]. Moreover, in males, both peak leg power and VO_2max_ have been linked with NHL performance [56] highlighting the distinct importance of both explosive strength and aerobic capacity. Consequently, off‐ice training typically integrates whole‐body strength, lower‐body plyometrics, and aerobic conditioning, which needs to be complemented by specific on‐ice work [52, 57]. In addition, modern monitoring enables on‐ice tracking of key performance indicators that can be used to individualize athletes' training [44, 46]. This conceptual distinction is reflected in Table 2, where aerobic capacity is framed primarily as a recovery and support capacity rather than a direct determinant of on‐ice work rate.

**TABLE 2:** Integrative summary of dominant performance determinants, key training priorities, and typical training characteristics across Olympic ice sports.

Sport/discipline	Dominant performance determinants	Key training priorities	Typical training characteristics	Sex‐specific considerations
Long‐track speed skating	Aerodynamic drag minimization; biomechanical efficiency (crouched posture); aerobic–anaerobic capacity; pacing ability	High‐volume endurance base; posture maintenance under fatigue; race‐specific intensity; lower‐body strength and core stability	~800–1100 h·year^−1^ total training; ~75%–80% low intensity; ~150–200 h on‐ice; extensive cross‐training (cycling)	Women show longer race times largely reflecting absolute power differences; technical determinants broadly similar across sexes
Short‐track speed skating	Start effectiveness; curve technique; tactical positioning; pacing variability; anaerobic power	Endurance base with mixed‐modal training; explosive starts; race‐simulation intervals; pack‐dynamic and decision‐making skills	Comparable total volume to long‐track (~800–1000 h·year^−1^); limited on‐ice volume due to local fatigue; high proportion of tactical on‐ice work	Core technical determinants similar; women may benefit more from deeper knee flexion; limited evidence for sex‐specific training adaptations
Bobsleigh	Start velocity; horizontal force application; sprint acceleration; aerodynamics during descent	Maximal and explosive lower‐body strength; sprint mechanics; sled‐push technique; start profiling	Training resembles sprint athletes; high emphasis on strength, power, and sprinting; limited endurance training	Men typically achieve higher start velocities due to greater absolute power; sled mass differs between sexes
Skeleton	Start velocity; sprint acceleration; load phase efficiency; steering precision	Sprint‐based strength and power training; plyometrics; start‐phase technical refinement	Off‐ice sprint and power training dominates; on‐ice start practice prioritized	Sex differences primarily reflect strength and power; relative determinants similar
Luge	Upper‐body explosive strength; paddling frequency; start‐phase coordination	Upper‐body maximal and explosive strength; start‐sequence technique; force–velocity optimization	High emphasis on upper‐body resistance training; simulators and refrigerated ramps used	Men generally faster due to greater upper‐body strength; technical structure comparable
Ice hockey	Repeated‐sprint ability; acceleration/deceleration; skating speed; aerobic recovery capacity	Speed endurance; repeated‐sprint training; lower‐body power; aerobic conditioning; on‐ice tactical drills	Mixed aerobic–anaerobic training; off‐ice strength and power complemented by high‐intensity on‐ice work	Females show slightly lower skating speeds; physiological determinants similar across sexes
Figure skating (singles, pairs, dance)	Jump take‐off velocity and angular momentum; technical consistency; balance; artistic execution	Power and plyometrics; unilateral strength; flexibility; technical repetition; program endurance	High on‐ice technical volume; substantial off‐ice conditioning (strength, ballet, flexibility)	Men more frequently execute higher‐rotation jumps; women often show higher angular velocity and flexibility
Curling	Delivery accuracy; perceptual–cognitive skills; sweeping force‐endurance; team coordination	Repeated delivery practice; sweeping strength‐endurance; perceptual training; tactical decision‐making	Moderate overall volume; emphasis on technical repetition and strength‐endurance rather than endurance	Limited evidence for major sex differences; men may generate greater sweeping force at matched cadence

### 5.2 Figure Skating

Figure skating involves choreographed ice routines blending complex technical skills (i.e., jumps, spins, lifts, footwork) with artistic interpretation under the ISU judging system [58]. Athlete performance is judged on both the execution of technical elements (base values and Grade of Execution, GOE) and overall program components (e.g., skating skills, choreography, musicality) (ISU). Competitive outcomes are largely dictated by the successful execution of high‐value technical elements, particularly multi‐rotation jumps in singles and pair disciplines, whose point values scale with rotation number and execution quality [59]. Successful jumps require sufficient take‐off velocities (vertical and angular), minimal in‐air moment of inertia via a compact posture to complete full rotation in air, and controlled landings [58, 60]. Other elements (spins, lifts, step sequences) reward speed, balance, flexibility, edge precision, and synchronization (in pairs/dance) [59]. Figure skating programs are physiologically exacting, blending high aerobic load with a substantial anaerobic contribution during technically demanding elements [61].

Elite skaters combine extensive on‐ice practice, focusing on element repetition and program run‐throughs for consistency and endurance, with targeted off‐ice conditioning [62]. Off‐ice training typically includes strength, power (including plyometrics), flexibility/mobility, endurance, and dance/ballet to develop the physical capacities underpinning on‐ice skills, with a key focus on the unilateral and asymmetrical force application required for jump take‐offs [58, 62].

Males, typically possessing greater absolute strength/power, more frequently execute higher‐scoring jumps (e.g., quadruples) with greater jump heights, up to 62 cm (about 2.03 ft) on ice (Unpublished data). Females, in turn, often demonstrate high levels of flexibility, facilitating varied spin positions [58, 62, 63], greater hip stability and endurance [64], and exhibit increased angular velocity during on‐ice jumps, up to 2000 degrees/s [65]. Sex‐specific differences in dynamic balance may also inform individualized conditioning and landing‐leg control [62]. In pairs and ice dance, differentiated roles (e.g., male lift/support vs. female flexibility/control) result in distinct physical demands, though artistic synchronicity is crucial for both partners [62]. Regardless of discipline, improvements in strength, power, and agility tend to increase among lower‐level skaters and plateau as the skater reaches the senior level, indicating the importance of athletes focusing on developing athleticism before reaching the senior, Olympic level [66]. As a result, figure skaters often participate in more training hours both on and off ice at younger ages and levels and slightly reduce training hours as their physical body develops to manage training loads.

### 5.3 Curling

Elite curling performance arises from the coupling of stone–ice physics with player technique and team management [67]. The stone curls because friction is stronger on one side than the other, thus narrowing the margin for steering [68], while active sweeping briefly reduces friction via ice heating [69]. Performance reflects the interaction of (1) individual capacities (delivery and sweeping technique, strength–endurance, balance, perceptual–cognitive skills), (2) team processes (strategy, communication, role execution, synchronized sweeping, real‐time decisions), and (3) environment and equipment (ice conditions, stone properties, release rotation/speed, broom characteristics, game context) [67]. From an individual standpoint, outcomes hinge on delivery quality and sweepers' ability to apply effective vertical load, both executed within the prevailing game context [67, 70, 71].

Within elite curling, success depends on delivery precision, perceptual acuity, and coordinated team strategy. The biomechanical, technical, and perceptual‐motor literature provides limited sex‐comparative evidence of large performance gaps, with some reports indicating small differences in mechanical outputs despite similar task execution [67]. Biomechanical field studies report broadly similar sweeping electromyography (EMG) profiles across sexes; at matched sweeping cadence, men often generate greater effective vertical load and consequently longer stone travel [71]. Collectively, available studies suggest any residual male advantage reflects differences in sweeping force capacity rather than clear disparities in perceptual–motor coordination or tactical proficiency, within the currently limited evidence base [67, 71].

Aligned with these determinants, elite training integrates upper‐body strength‐endurance for sweeping, interval bouts that replicate sweeping demands and repeated delivery sessions to refine shot‐making consistency and spatial accuracy [72]. The skills of release speed and line control are highly trainable, and recent applied research shows that stroboscopic vision training improves duration judgment and speed control, whereas temporal‐feedback training yields larger accuracy gains over 4 weeks [73].

## 6 Conclusion

This review has demonstrated that Olympic ice sports encompass a broad spectrum of performance determinants shaped by the interaction of biomechanics, physiology, and technical execution. While volume‐driven disciplines such as long‐ and short‐track speed skating are comparatively well characterized, exposure‐driven gravity sports and arena‐based ice sports still lack comprehensive and systematic scientific coverage. The existing literature is fragmented, often discipline‐specific, and rarely comparable across studies, limiting knowledge transfer and best‐practice development.

For synthesis and clarity, Table 2 provides an integrative summary of the dominant performance determinants, key training priorities, and typical training characteristics across Olympic ice sports, structured according to the three performance‐demand categories proposed in this review (high‐volume gliding, exposure‐driven gravity, and arena‐based sports) (Table 2).

Where the available evidence permits, sex‐specific considerations are explicitly indicated; where data are limited or inconsistent, this is clearly acknowledged.

## 7 Perspectives

Future research should adopt a more explicit, discipline‐sensitive agenda that reflects the uneven distribution of empirical evidence across Olympic ice sports. Arena‐based disciplines such as curling and figure skating, as well as exposure‐driven sports such as luge, remain particularly underrepresented with respect to systematic analyses of training structure, load monitoring, and longitudinal performance development.

To operationalize these needs, Table 3 summarizes discipline‐specific future research priorities alongside corresponding actionable insights for athlete preparation. This table explicitly links current evidence gaps with practical implications for training design, monitoring, and performance optimization across high‐volume gliding, exposure‐driven gravity, and arena‐based sports. By pairing research questions with applied considerations, Table 3 is intended to support both hypothesis‐driven research planning and evidence‐informed coaching practice.

**TABLE 3:** Discipline‐specific future research priorities and actionable insights for athlete preparation.

Discipline group	Future research priorities	Actionable insights for practice
High‐volume gliding sports (long‐ and short‐track speed skating)	Longitudinal links between posture durability, muscle oxygenation, and performance decline Sex‐specific recovery kinetics and training‐load tolerance Training and transfer of drafting and pack‐dynamic skills	Prioritize endurance durability under sport‐specific posture Use mixed‐modal endurance training to manage musculoskeletal load Integrate race‐simulation and tactical decision‐making sessions
Exposure‐driven gravity sports (bobsleigh, skeleton, luge)	Transfer mechanisms between off‐ice power tests and on‐ice start performance Sex‐specific strength–power profiling relative to sled mass and inertia Fatigue, injury risk, and recovery during high‐volume start training	Emphasize sprint‐ and power‐based preparation Apply start‐velocity profiling for individualized feedback Use endurance training primarily to support training tolerance and recovery
Arena‐based sports—Ice hockey	Dose–response relationships between aerobic conditioning and repeated‐sprint ability Sex‐specific adaptations to intermittent high‐intensity loads Integration of external (on‐ice tracking) and internal load metrics	Balance aerobic conditioning with repeated‐sprint and speed‐endurance training Individualize training by position and shift demands Use monitoring to guide recovery during congested competition schedules
Arena‐based sports—Figure skating	Effects of neuromuscular fatigue on jump execution and landing mechanics Sex‐ and discipline‐specific load management across the season Long‐term athlete development and injury risk trajectories	Prioritize power development and landing control over excessive endurance volume Use endurance training to support program repeatability Carefully manage training load during growth and maturation
Arena‐based sports—Curling	Determinants of sweeping strength–endurance and effectiveness Transfer of perceptual–cognitive and vision training to performance Sex‐comparative studies under elite match conditions	Focus on precision, sweeping strength–endurance, and decision‐making Integrate perceptual and vision‐based training tools Apply aerobic conditioning conservatively as a support capacity

Progress in these areas depends on improved data harmonization, interdisciplinary collaboration, and the targeted use of emerging technologies. Wearable sensors, on‐ice tracking, AI‐based video analysis, instrumented force measurements, and computational modeling offer high‐resolution, ecologically valid insights into technique, load distribution, and fatigue. When integrated with established physiological and biomechanical monitoring in longitudinal and sex‐inclusive designs, these approaches can strengthen mechanistic understanding and support transferable, evidence‐based performance models.

Across disciplines, several crosscutting training priorities emerge (Tables 2 and 3): standardized documentation of training structure to clarify dose–response relationships; improved understanding of peaking and tapering, particularly with respect to sex‐specific recovery and competition demands; and greater emphasis on individualized recovery strategies to support sustainable performance and reduce injury and illness risk.

In addition, climate change and sustainability considerations are becoming increasingly relevant for Olympic ice sports, with direct implications for training environments, competition logistics, and venue management. Rising temperatures and climatic variability affect ice quality, the reliability of outdoor facilities, and the availability of suitable training venues, increasing reliance on artificial ice production, alternative surface materials, and long‐distance travel. These developments raise environmental and economic concerns and may influence training consistency, athlete health, and competitive equity. Future research and strategic planning should therefore consider sustainable approaches to facility design, ice and surface technologies, travel logistics, and training periodization, aligning performance optimization with long‐term ecological responsibility.

Ultimately, a coordinated effort among sport scientists, coaches, and federations is required to translate empirical findings into applied frameworks that reflect the distinct performance demands of volume‐, exposure‐, and arena‐driven ice sports. Together, Tables 2 and 3 provide complementary synthesis tools—one summarizing current determinants and training characteristics, and the other outlining forward‐looking research and practice priorities—to support evidence‐based and sex‐inclusive athlete preparation as the Olympic movement progresses toward Milano‐Cortina 2026 and beyond.

## Funding

The authors have nothing to report.

## Conflicts of Interest

The authors declare no conflicts of interest.

## Data Availability

The authors have nothing to report.

## References

[1] J. J. de Koning , F. C. Bakker , G. de Groot , and G. J. van Ingen Schenau , “Longitudinal Development of Young Talented Speed Skaters: Physiological and Anthropometric Aspects,” Journal of Applied Physiology (1985) 77, no. 5 (1994): 2311–2317, 10.1152/jappl.1994.77.5.2311. 7868450

[2] C. Foster , J. J. deKoning , F. Hettinga , et al., “Effect of Competitive Distance on Energy Expenditure During Simulated Competition,” International Journal of Sports Medicine 25, no. 3 (2004): 198–204, c 15088244 10.1055/s-2003-45260

[3] S. G. P. Menting , B. C. Huijgen , M. J. Konings , F. J. Hettinga , and M. T. Elferink‐Gemser , “Pacing Behavior Development of Youth Short‐Track Speed Skaters: A Longitudinal Study,” Medicine and Science in Sports and Exercise 52, no. 5 (2020): 1099–1108. 10.1249/MSS.0000000000002239. 31815834

[4] I. K. Stoter , B. R. MacIntosh , J. R. Fletcher , S. Pootz , I. Zijdewind , and F. J. Hettinga , “Pacing Strategy, Muscle Fatigue, and Technique in 1500‐m Speed‐Skating and Cycling Time Trials,” International Journal of Sports Physiology and Performance 11, no. 3 (2016): 337–343, cc 26263372 10.1123/ijspp.2014-0603

[5] I. K. Stoter , F. J. Hettinga , E. Otten , C. Visscher , and M. T. Elferink‐Gemser , “Changes in Technique Throughout a 1500‐m Speed Skating Time‐Trial in Junior Elite Athletes: Differences Between Sexes, Performance Levels and Competitive Seasons,” PLoS One 15, no. 8 (2020): e0237331, 10.1371/journal.pone.0237331. 32822398 PMC7446824

[6] A. Hext , F. J. Hettinga , and C. McInerney , “Tactical Positioning in Short‐Track Speed Skating: The Utility of Race‐Specific Athlete‐Opponent Interactions,” European Journal of Sport Science 23, no. 5 (2023): 693–702, 10.1080/17461391.2022.2069513. 35446752

[7] G. J. van Ingen Schenau , J. J. de Koning , and G. de Groot , “A Simulation of Speed Skating Performances Based on a Power Equation,” Medicine and Science in Sports and Exercise 22, no. 5 (1990): 718–728, 10.1249/00005768-199010000-00026. 2233213

[8] J. J. de Koning , C. Foster , J. Lampen , F. Hettinga , and M. F. Bobbert , “Experimental Evaluation of the Power Balance Model of Speed Skating,” Journal of Applied Physiology (1985) 98, no. 1 (2005): 227–233, 10.1152/japplphysiol.01095.2003. 15591304

[9] D. A. Noordhof , C. Foster , M. J. Hoozemans , and J. J. de Koning , “Changes in Speed Skating Velocity in Relation to Push‐Off Effectiveness,” International Journal of Sports Physiology and Performance 8, no. 2 (2013): 188–194, 10.1123/ijspp.8.2.188. 23428491

[10] M. J. Konings , M. T. Elferink‐Gemser , I. K. Stoter , D. van der Meer , E. Otten , and F. J. Hettinga , “Performance Characteristics of Long‐Track Speed Skaters: A Literature Review,” Sports Medicine 45, no. 4 (2015): 505–516, 10.1007/s40279-014-0298-z. 25547998

[11] D. P. Born , C. Zinner , B. Herlitz , K. Richter , H. C. Holmberg , and B. Sperlich , “Muscle Oxygenation Asymmetry in Ice Speed Skaters: Not Compensated by Compression,” International Journal of Sports Physiology and Performance 9, no. 1 (2014): 58–67, 10.1123/ijspp.2012-0210. 23239684

[12] C. Foster , K. W. Rundell , A. C. Snyder , et al., “Evidence for Restricted Muscle Blood Flow During Speed Skating,” Medicine and Science in Sports and Exercise 31, no. 10 (1999): 1433–1440, 10.1097/00005768-199910000-00012. 10527316

[13] F. J. Hettinga , M. J. Konings , and C. E. Cooper , “Differences in Muscle Oxygenation, Perceived Fatigue and Recovery Between Long‐Track and Short‐Track Speed Skating,” Frontiers in Physiology 7 (2016): 619, 10.3389/fphys.2016.00619. 28018244 PMC5156719

[14] M. J. Konings , O. S. Noorbergen , D. Parry , and F. J. Hettinga , “Pacing Behavior and Tactical Positioning in 1500‐m Short‐Track Speed Skating,” International Journal of Sports Physiology and Performance 11, no. 1 (2016): 122–129, 10.1123/ijspp.2015-0137. 26062042

[15] O. Elfmark , L. M. Bardal , L. Oggiano , and H. Myklebust , “Aerodynamic Interaction Between Two Speed Skaters Measured in a Closed Wind Tunnel,” International Journal of Sport and Health Sciences 13 (2019): 191–201.

[16] F. A. P. van den Brandt , S. G. P. Menting , F. J. Hettinga , and M. T. Elferink‐Gemser , “Drafting in Long‐Track Speed Skating Team Pursuit on the Ice Rink,” Journal of Sports Sciences 41, no. 5 (2023): 456–462, 10.1080/02640414.2023.2223034. 37330667

[17] F. A. van den Brandt , S. G. Menting , B. C. Huijgen , F. J. Hettinga , and M. T. Elferink‐Gemser , “Do Athletes and Coaches Do What They Know? A Mixed‐Method Survey on Beliefs and Attitudes on Drafting in Endurance Sports,” International Journal of Sports Science and Coaching (2025): 10.1177/17479541251386161.

[18] J. Orie , N. Hofman , L. A. Meerhoff , and A. Knobbe , “Training Distribution in 1500‐m Speed Skating: A Case Study of an Olympic Gold Medalist,” International Journal of Sports Physiology and Performance 16, no. 1 (2021): 149–153, 10.1123/ijspp.2019-0544. 33004683

[19] O. Sandbakk , E. Tonnessen , S. B. Sandbakk , T. Losnegard , S. Seiler , and T. Haugen , “Best‐Practice Training Characteristics Within Olympic Endurance Sports as Described by Norwegian World‐Class Coaches,” Sports Medicine—Open 11, no. 1 (2025): 45, 10.1186/s40798-025-00848-3. 40278987 PMC12031707

[20] B. Sperlich , M. Matzka , and H. C. Holmberg , “The Proportional Distribution of Training by Elite Endurance Athletes at Different Intensities During Different Phases of the Season,” Frontiers in Sports and Active Living 5 (2023): 1258585, 10.3389/fspor.2023.1258585. 37964776 PMC10641476

[21] L. Sun , T. Guo , F. Liu , and K. Tao , “Champion Position Analysis in Short Track Speed Skating Competitions From 2007 to 2019,” Frontiers in Psychology 12 (2021): 760900, 10.3389/fpsyg.2021.760900. 34950087 PMC8689186

[22] M. J. Konings and F. J. Hettinga , “Pacing Decision Making in Sport and the Effects of Interpersonal Competition: A Critical Review,” Sports Medicine (Auckland, N.Z.) 48, no. 8 (2018): 1829–1843, 10.1007/s40279-018-0937-x. 29799094

[23] O. S. Noorbergen , M. J. Konings , D. Micklewright , M. T. Elferink‐Gemser , and F. J. Hettinga , “Pacing Behavior and Tactical Positioning in 500‐ and 1000‐m Short‐Track Speed Skating,” International Journal of Sports Physiology and Performance 11, no. 6 (2016): 742–748, 10.1123/ijspp.2015-0384. 26641204

[24] A. Hext , F. J. Hettinga , and C. McInerney , “Tactical Positioning Behaviours in Short‐Track Speed Skating: A Static and Dynamic Sequence Analysis,” Journal of Sports Sciences 41, no. 8 (2023): 727–735, 10.1080/02640414.2023.2238162. 37496326

[25] E. van der Kruk , M. M. Reijne , B. de Laat , and D. Veeger , “Push‐Off Forces in Elite Short‐Track Speed Skating,” Sports Biomechanics 18, no. 5 (2019): 527–538, 10.1080/14763141.2018.1441898. 29847206

[26] J. Claudel and J. Clement , “Analysing Short‐Track Speed Skating Performance Factors,” Sports Biomechanics 24, no. 12 (2025): 3739–3752, 10.1080/14763141.2025.2557400. 40981496

[27] C. M. Hesford , S. Laing , M. Cardinale , and C. E. Cooper , “Effect of Race Distance on Muscle Oxygenation in Short‐Track Speed Skating,” Medicine and Science in Sports and Exercise 45, no. 1 (2013): 83–92, 10.1249/MSS.0b013e31826c58dd. 22895375

[28] S. Deguire , G. N. Sandford , and F. Bieuzen , “Anaerobic Speed Reserve and Performance Relationships Between International and World‐Class Short‐Track Speed Skating,” International Journal of Sports Physiology and Performance 18, no. 10 (2023): 1196–1205, 10.1123/ijspp.2023-0055. 37536677

[29] T. Méline , L. Mathieu , F. Borrani , R. Candau , and A. M. Sanchez , “Systems Model and Individual Simulations of Training Strategies in Elite Short‐Track Speed Skaters,” Journal of Sports Sciences 37, no. 3 (2019): 347–355. 30071185 10.1080/02640414.2018.1504375

[30] P. Gimenez , E. Chicoine , D. Amarantini , F. D. Maso , and J. Tremblay , “Unilateral Conditioning Contractions Enhance Power Output in Elite Short Track Speed Skaters,” Sports Medicine International Open 2, no. 6 (2018): E185–E190, 10.1055/a-0770-4699. 30539137 PMC6277240

[31] S. Felser , M. Behrens , S. Fischer , et al., “Relationship Between Strength Qualities and Short Track Speed Skating Performance in Young Athletes,” Scandinavian Journal of Medicine & Science in Sports 26, no. 2 (2016): 165–171, 10.1111/sms.12429. 25683194

[32] F. Borrani , R. Solsona , R. Candau , T. Méline , and A. M. Sanchez , “Modelling Performance With Exponential Functions in Elite Short‐Track Speed Skaters,” Journal of Sports Sciences 39, no. 20 (2021): 2378–2385. 34058952 10.1080/02640414.2021.1933351

[33] N. Bullock , W. G. Hopkins , D. T. Martin , and F. E. Marino , “Characteristics of Performance in Skeleton World Cup Races,” Journal of Sports Sciences 27, no. 4 (2009): 367–372. 19235005 10.1080/02640410802613425

[34] S. L. Colyer , K. A. Stokes , J. L. J. Bilzon , and A. I. T. Salo , “Skeleton Sled Velocity Profiles: A Novel Approach to Understand Critical Aspects of the Elite Athletes' Start Phases,” Sports Biomechanics 17, no. 2 (2018): 168–179, 10.1080/14763141.2016.1261183. 28632062

[35] M. Zedler , B. Braunstein , W. Potthast , and J. P. Goldmann , “Biomechanics of the Bobsleigh Push Phase,” Journal of Sports Sciences 43, no. 4 (2025): 360–369, 10.1080/02640414.2025.2458983. 39873353

[36] S. L. Colyer , S. P. Roberts , J. B. Robinson , et al., “Detecting Meaningful Body Composition Changes in Athletes Using Dual‐Energy X‐Ray Absorptiometry,” Physiological Measurement 37, no. 4 (2016): 596–609, 10.1088/0967-3334/37/4/596. 27027548

[37] S. L. Colyer , K. A. Stokes , J. L. Bilzon , M. Cardinale , and A. I. Salo , “Physical Predictors of Elite Skeleton Start Performance,” International Journal of Sports Physiology and Performance 12, no. 1 (2017): 81–89, 10.1123/ijspp.2015-0631. 27140284

[38] B. W. Crossland , J. E. Hartman , J. L. Kilgore , M. J. Hartman , and J. M. Kaus , “Upper‐Body Anthropometric and Strength Measures and Their Relationship to Start Time in Elite Luge Athletes,” Journal of Strength and Conditioning Research 25, no. 10 (2011): 2639–2644, 10.1519/JSC.0b013e318207ed7a. 21873904

[39] L. Demuth , C. Patterson , F. F. Locker , and C. Raschner , “Force‐Velocity Profiling in Elite Luge Athletes During the Simulated Luge Start,” Current Issues in Sport Science (CISS) 9, no. 4 (2024): 20.

[40] W. A. Sands , L. S. Smith , D. M. Kivi , et al., “Anthropometric and Physical Abilities Profiles: US National Skeleton Team,” Sports Biomechanics 4, no. 2 (2005): 197–214, 10.1080/14763140508522863. 16138657

[41] S. L. Colyer , K. A. Stokes , J. L. J. Bilzon , D. Holdcroft , and A. I. T. Salo , “Training‐Related Changes in Force‐Power Profiles: Implications for the Skeleton Start,” International Journal of Sports Physiology and Performance 13, no. 4 (2018): 412–419, 10.1123/ijspp.2017-0110. 28872389

[42] N. Bullock , J. P. Gulbin , D. T. Martin , A. Ross , T. Holland , and F. Marino , “Talent Identification and Deliberate Programming in Skeleton: Ice Novice to Winter Olympian in 14 Months,” Journal of Sports Sciences 27, no. 4 (2009): 397–404, 10.1080/02640410802549751. 19191166

[43] J. F. Vigh‐Larsen and M. Mohr , “The Physiology of Ice Hockey Performance: An Update,” Scandinavian Journal of Medicine and Science in Sports 34, no. 1 (2024): e14284, 10.1111/sms.14284. 36517860

[44] A. S. D. Gamble , J. L. Bigg , D. L. E. Nyman , and L. L. Spriet , “Local Positioning System‐Derived External Load of Female and Male Varsity Ice Hockey Players During Regular Season Games,” Frontiers in Physiology 13 (2022): 831723, 10.3389/fphys.2022.831723. 35283770 PMC8914021

[45] E. Lignell , D. Fransson , P. Krustrup , and M. Mohr , “Analysis of High‐Intensity Skating in Top‐Class Ice Hockey Match‐Play in Relation to Training Status and Muscle Damage,” Journal of Strength and Conditioning Research 32, no. 5 (2018): 1303–1310, 10.1519/JSC.0000000000001999. 28557852

[46] A. Douglas , K. Johnston , J. Baker , M. A. Rotondi , V. K. Jamnik , and A. K. Macpherson , “On‐Ice Measures of External Load in Relation to Match Outcome in Elite Female Ice Hockey,” Sports (Basel, Switzerland) 7, no. 7 (2019): 173, 10.3390/sports7070173. 31315209 PMC6681036

[47] J. F. Vigh‐Larsen , G. Ermidis , V. Rago , et al., “Muscle Metabolism and Fatigue During Simulated Ice Hockey Match‐Play in Elite Players,” Medicine and Science in Sports and Exercise 52, no. 10 (2020): 2162–2171, 10.1249/MSS.0000000000002370. 32496739

[48] J. F. Vigh‐Larsen , N. Ortenblad , J. Nielsen , O. Emil Andersen , K. Overgaard , and M. Mohr , “The Role of Muscle Glycogen Content and Localization in High‐Intensity Exercise Performance: A Placebo‐Controlled Trial,” Medicine and Science in Sports and Exercise 54, no. 12 (2022): 2073–2086, 10.1249/MSS.0000000000003002. 35868015

[49] M. Glaister , “Multiple Sprint Work: Physiological Responses, Mechanisms of Fatigue and the Influence of Aerobic Fitness,” Sports Medicine 35, no. 9 (2005): 757–777, 10.2165/00007256-200535090-00003. 16138786

[50] J. F. Vigh‐Larsen , M. T. Haverinen , J. Panduro , et al., “On‐Ice and Off‐Ice Fitness Profiles of Elite and U20 Male Ice Hockey Players of Two Different National Standards,” Journal of Strength and Conditioning Research 34, no. 12 (2020): 3369–3376, 10.1519/JSC.0000000000003836. 33009345

[51] J. F. Vigh‐Larsen , J. H. Beck , A. Daasbjerg , et al., “Fitness Characteristics of Elite and Subelite Male Ice Hockey Players: A Cross‐Sectional Study,” Journal of Strength and Conditioning Research 33, no. 9 (2019): 2352–2360, 10.1519/JSC.0000000000003285. 31343551

[52] M. Boland , K. Delude , and E. M. Miele , “Relationship Between Physiological Off‐Ice Testing, On‐Ice Skating, and Game Performance in Division I Female Ice Hockey Players,” Journal of Strength and Conditioning Research 33, no. 6 (2019): 1619–1628, 10.1519/JSC.0000000000002265. 29016475

[53] K. M. Gilenstam , K. Thorsen , and K. B. Henriksson‐Larsen , “Physiological Correlates of Skating Performance in Women's and Men's Ice Hockey,” Journal of Strength and Conditioning Research 25, no. 8 (2011): 2133–2142, 10.1519/JSC.0b013e3181ecd072. 21785292

[54] M. A. Czeck , E. J. Roelofs , C. Dietz , T. A. Bosch , and D. R. Dengel , “Body Composition and On‐Ice Skate Times for National Collegiate Athletic Association Division I Collegiate Male and Female Ice Hockey Athletes,” Journal of Strength and Conditioning Research 36, no. 1 (2022): 187–192, 10.1519/JSC.0000000000004175. 34941612

[55] J. J. de Koning , R. Thomas , M. Berger , G. de Groot , and G. J. van Ingen Schenau , “The Start in Speed Skating: From Running to Gliding,” Medicine and Science in Sports and Exercise 27, no. 12 (1995): 1703–1708. 8614329

[56] J. N. Cohen , K. M. A. Thompson , V. K. Jamnik , N. Gledhill , and J. F. Burr , “Relationship of Fitness Combine Results and National Hockey League Performance: A 25‐Year Analysis,” International Journal of Sports Physiology and Performance 17, no. 6 (2022): 908–916, 10.1123/ijspp.2021-0317. 35245896

[57] L. B. Ransdell , T. M. Murray , and Y. Gao , “Off‐Ice Fitness of Elite Female Ice Hockey Players by Team Success, Age, and Player Position,” Journal of Strength and Conditioning Research 27, no. 4 (2013): 875–884, 10.1519/JSC.0b013e3182651fd2. 22739329

[58] D. L. King , “Performing Triple and Quadruple Figure Skating Jumps: Implications for Training,” Canadian Journal of Applied Physiology 30, no. 6 (2005): 743–753, 10.1139/h05-153. 16485524

[59] S. Hirosawa , M. Watanabe , and Y. Aoki , “Determinant Analysis and Developing Evaluation Indicators of Grade of Execution Score of Double Axel Jump in Figure Skating,” Journal of Sports Sciences 40, no. 4 (2022): 470–481, 10.1080/02640414.2021.1997407. 34781855

[60] M. Yamaguchi and S. Sakurai , “Comparisons of Angular Momentum at Takeoff in Six Types of Jumps in Women's Figure Skating,” Frontiers in Sports and Active Living 7 (2025): 1597598, 10.3389/fspor.2025.1597598. 40948483 PMC12426178

[61] M. Kjaer and B. Larsson , “Physiological Profile and Incidence of Injuries Among Elite Figure Skaters,” Journal of Sports Sciences 10, no. 1 (1992): 29–36, 10.1080/02640419208729904. 1556775

[62] L. V. Slater , M. Vriner , P. Zapalo , K. Arbour , and J. M. Hart , “Difference in Agility, Strength, and Flexibility in Competitive Figure Skaters Based on Level of Expertise and Skating Discipline,” Journal of Strength and Conditioning Research 30, no. 12 (2016): 3321–3328, 10.1519/JSC.0000000000001452. 27100316

[63] S. Hirosawa , “Kinematic Considerations for Achieving the Quadruple Axel Jump: Comparison With Triple Axel Jumps Among World‐Class Figure Skaters Using Tracking Data,” Sports Biomechanics (2025): 1–12, 10.1080/14763141.2025.2464787. 39950336

[64] B. Webb , J. H. Kenning , A. Guzman , L. Slater , and L. C. Mangum , “The Lumbopelvic‐Hip Complex Contribution During Lower Extremity Screening Tests in Elite Figure Skaters,” Journal of Athletic Training 57, no. 6 (2022): 581–585, 10.4085/1062-6050-0373.21. 35969665 PMC9387377

[65] L. S. Hannigan , “Female Figure Skaters Demonstrate Greater Peak Angular Velocity Compared to Male Figure Skaters: 645,” Medicine & Science in Sports & Exercise 57, no. 10S (2025): 210–211.39283230

[66] J. P. Cruz , M. Vriner , L. C. Mangum , and L. Slater , “Longitudinal Changes in Athletic Performance in Competitive Figure Skaters,” Journal of Science in Sport and Exercise 3, no. 4 (2021): 332–339.

[67] E. Zacharias , N. Robak , and S. Passmore , “An Examination of Studies Related to the Sport of Curling: A Scoping Review,” Frontiers in Sports and Active Living 6 (2024): 1291241, 10.3389/fspor.2024.1291241. 38414637 PMC10898248

[68] J. Murata , “Study of Curling Mechanism by Precision Kinematic Measurements of Curling Stone's Motion,” Scientific Reports 12, no. 1 (2022): 15047, 10.1038/s41598-022-19303-4. 36057647 PMC9440934

[69] J. L. Bradley , “The Sports Science of Curling: A Practical Review,” Journal of Sports Science and Medicine 8, no. 4 (2009): 495–500. 24149588 PMC3761524

[70] L. Hua , Y. Zhu , G. Bi , M. Zhu , and Y. Diao , “Effects of Sliding Techniques on Lower Limb Biomechanics and Muscle Synergy During Curling Delivery: A Focus on Joint Kinetics and Muscle Coordination,” Frontiers in Human Neuroscience 19 (2025): 1587118, 10.3389/fnhum.2025.1587118. 40654565 PMC12245892

[71] T. W. Kim , S. C. Lee , S. K. Kil , S. H. Choi , and Y. G. Song , “A Case Study on Curling Stone and Sweeping Effect According to Sweeping Conditions,” International Journal of Environmental Research and Public Health 18, no. 2 (2021): 833, 10.3390/ijerph18020833. 33478079 PMC7835804

[72] D. G. Behm , “Periodized Training Program of the Canadian Olympic Curling Team,” Strength & Conditioning Journal 29, no. 3 (2007): 24–31.

[73] T. Li , X. Wang , Z. Wu , and Y. Liang , “The Effect of Stroboscopic Vision Training on the Performance of Elite Curling Athletes,” Scientific Reports 14, no. 1 (2024): 31730, 10.1038/s41598-024-82685-0. 39738499 PMC11685645

